# A framework for the recursive risk-ranking of foodborne zoonotic threats along food supply chains

**DOI:** 10.1016/j.onehlt.2026.101415

**Published:** 2026-04-15

**Authors:** Sascha Al Dahouk, Gilberto Montibeller

**Affiliations:** a German Federal Institute for Risk Assessment, Department of Biological Safety, Berlin, Germany; b Robert Koch Institute, Department 1 – Infectious Diseases, Berlin, Germany; c RWTH Aachen University Hospital, Department of Internal Medicine III, Aachen, Germany; d University of Bristol Business School, University of Bristol, Bristol, UK; e KFUPM Business School, KFUPM, Dhahran, Saudi Arabia

**Keywords:** Food safety risk analysis, Foodborne threats, Food supply chains, National food security, Health security decision analysis, Risk-based decision making, Recursive risk-ranking

## Abstract

The threat of foodborne disease outbreaks poses a significant challenge to food safety and public health. Novel pathogens are emerging, and old diseases are re-emerging in complex and long food supply chains that increase the number of pathways for food contamination. Hence, there is growing recognition of the importance of risk prioritization for food safety systems and for supporting risk-based decision making. In this paper, we propose a framework for the recursive risk-ranking of foodborne zoonotic threats along food supply chains, which considers both the multidimensional nature of the impact assessment and the probabilistic estimates of the prevalence of zoonotic pathogens in food supply chains. Although the entire food chain *from stable to table* must be assessed and controlled to eradicate zoonotic diseases in animal reservoirs and prevent human infections (following the *One Health* concept), we focus on foodstuffs as sources of pathogens because they directly lead to exposure through consumption. The framework allows iterative application of the evaluation model to prioritize threats and update these priorities as new evidence emerges, while also enabling refinement of the model as the decision context evolves. We assessed the framework at the German Federal Institute for Risk Assessment with encouraging results. We discuss our results and the potential benefits of such recursive assessments for managing supply-chain food risks and enhancing national and global food safety.

## Highlights

• Proposes a framework to prioritize foodborne zoonotic risks in food supply chains.
• Identifies recursively key zoonotic threats in complex food supply chains.
• Integrates impact assessment with pathogen prevalence data.
• Assesses the framework using expert input and data from German risk assessors.
• Aims to enhance food supply chain safety following the One Health approach.

## 1 Introduction

The threat of foodborne disease outbreaks poses a significant challenge to food safety and public health. Novel pathogens are emerging, and old diseases are re-emerging in response to changing driving forces, such as climate change [1], as well as complex and long food supply chains that increase the number of pathways for food contamination [2]. These health threats add complexity to food supply chain management [3].

Due to the scarcity of resources in the food sector and the increasing consumer demand for affordable, healthy and safe food, resources must be allocated to the most efficient and effective measures to combat foodborne zoonotic risks along food supply chains. In addition, there is an increasing recognition of the importance of risk prioritizations for food safety systems [4] and for supporting risk-based decision making [5], [6], [7], given the multi-dimensional nature and complexity of these assessments [8]. While there are well-established frameworks for One Health community-based prioritization of zoonotic diseases, which have been successfully applied in practice [9], [10], [11], these frameworks are not focused on food supply chains, nor can they be recursively used to risk-rank emerging or re-emerging threats. The key research question addressed in our study is how risk prioritization for such threats along supply chains can be better supported.

In this paper, we propose a framework for risk-ranking foodborne zoonotic threats along food supply chains that considers both the multidimensional nature of impact assessment and probabilistic estimates of zoonotic pathogen prevalence. We assessed the framework at the German Federal Institute for Risk Assessment (Bundesinstitut für Risikobewertung, BfR) with encouraging results. The next section provides a brief literature review on the prioritization of such threats. The suggested framework is described in the subsequent section, followed by the case study results and a discussion of policy implications.

## 2 Literature review: prioritization of foodborne zoonotic threats

In this section, we briefly discuss current efforts to prioritize foodborne zoonotic threats, highlight the requirements for such prioritization, and identify the limitations of existing assessments. Although food safety interventions are multi-dimensional, risk-benefit assessments mainly target health impacts (and may consider countermeasures taken in surveillance and control of food contamination), but lack the evaluation of other factors, with social value trade-offs often being neglected [12].

Given the inherently multidimensional nature of this type of prioritization, Multi-Criteria Decision Analysis (MCDA) has already improved the identification, reporting, and assessment of emerging threats to both animal and public health [2], [13], [14], [15], [16]. Hence, this methodology can be applied to prioritize foodborne zoonotic threats [6] in a structured, systematic, and consistent way that may help decision-makers to improve risk monitoring and preparedness [17]. However, proposals to extend this food risk ranking assessment to consider multiple criteria have typically employed poor modeling practices [18].

In food safety, MCDA has been applied to various types of evaluation related to food supply management [19], to risk ranking of zoonoses [20], and to intervention measures to reduce the risk of a pathogen in a food product [21]. The major strength of MCDA is its ability to combine different types of impacts and to represent judgments from decision-makers (valuations) and experts (estimates of possible impacts) in a coherent way [12]. Multi-criteria decision analysis may consequently support policymakers in identifying the best solutions to food safety problems and in making reasoned decisions [22].

Prevalence data on pathogens in particular food products are often gathered from passive and active surveillance systems and outbreak databases. However, the attributable risk is typically estimated only for foodstuffs considered to pose a high risk [23] and for very specific pathogen-food matrix combinations. Furthermore, the probability of infection is linked not only to the occurrence within a single major transmission pathway but also to the overall prevalence in foodstuffs, consumers' consumption habits, and the pathogen's virulence, among other factors.

To introduce efficient and targeted preventive measures at the regional or national level promptly and reduce the impact of zoonotic pathogens along food supply chains, high-priority diseases should be identified regularly. In this way, food safety controls can target specific pathogens or food supply chains. The recursive nature of these decisions calls for the development of risk management systems to comprehensively assess emerging health threats along food supply chains [2]. To our knowledge, however, such recursive prioritization approaches have not yet been proposed in the food safety literature.

## 3 Methodology: the recursive risk-ranking framework

**Fig. 1:**
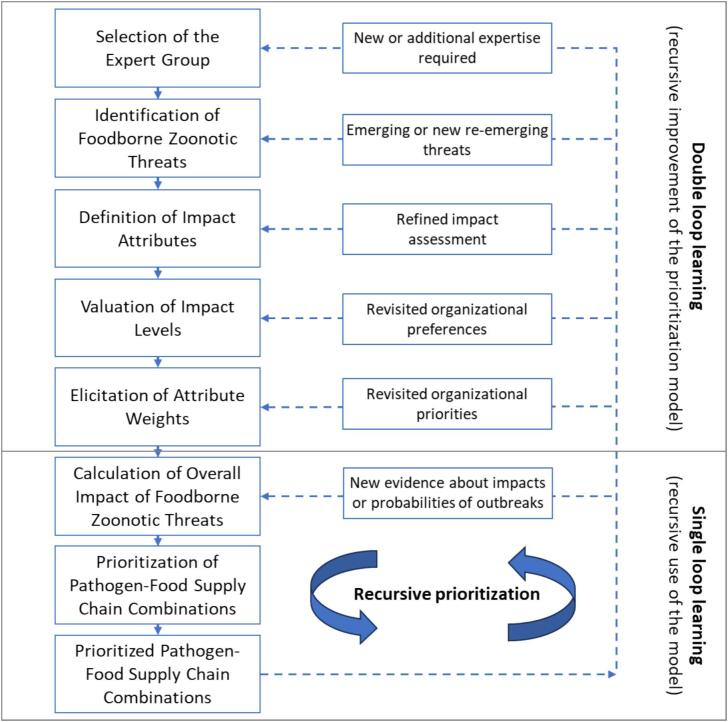
The recursive framework for risk-ranking foodborne zoonotic threats along food supply chains.

Our proposed framework for the risk ranking of foodborne zoonotic threats along food supply chains (see Fig. 1) is rooted in Health Decision Analysis [17], [24]. The individual steps of the framework are shown on the left-hand side of Fig. 1. The valuation of impacts employs Multi-Attribute Value Theory [25], which has been applied in several prioritizations of health threats [14], [26], [27]; it has a strong axiomatic basis, well-developed protocols for preference elicitation, and can be adequately combined with probabilistic events [18].

The impact assessment uses constructed attributes [28]. This approach also accelerates urgent decisions when dealing with emerging threats. In the evaluation model, we separated value elicitation from the final risk ranking process [29]. Specifically, we first elicited preferences (ranking and dis-value scoring of attribute levels, as well as swing weighting of impact attributes) independent of the specific foodborne zoonotic threats under consideration to avoid motivational biases [30] that may otherwise over-emphasize a particular threat [30], [31].

The elicitation of trade-offs among multiple impacts is key for reasoned decisions with conflicting objectives, which are frequent in food-related decision contexts [32]. We elicited the prioritization of impact attributes from experts using the swing weighting elicitation protocol [12]. This protocol requires the consideration of both the relevance of the impact and the range of impacts, thus minimizing the range-insensitivity bias [30] that could distort the valuation.

We assigned estimated probabilities to the assessed impacts by using information on the prevalence of the foodborne zoonotic threats, thereby avoiding the equal-probability assumption often employed in multi-criteria prioritization of health threats [33]. Although expert elicitation can be useful when data are lacking, expert judgments may be subject to bias [34]. High-risk food may lead to an overestimation of the importance of a food vehicle in sporadic cases, which is why expert and outbreak attribution estimates may vary [35]. In our model, we decomposed the valuation of impacts and the estimation of occurrence probabilities to minimize this motivational bias.

The framework enables *recursive* prioritization of threats. Specifically, as illustrated on the right-hand side of Fig. 1, the evaluation model enables the prioritization of threats based on their multiple impacts and probabilities of occurrence; as new evidence about impacts or probabilities becomes available, the model's recursive use enables the re-prioritization of threats (single-loop learning). The model itself can be improved, given revisited organizational priorities and preferences, refined impact assessments, emerging or re-emerging threats, or the need for new or additional expertise (double-loop learning). We illustrate the use of the framework at the German Federal Institute for Risk Assessment (BfR) in this section and reflect on its potential for recursive prioritizations in the discussion section.

### 3.1 Selection of the expert group

To facilitate and accelerate the development of the prioritization model, we selected a small but highly heterogeneous group of experts and risk assessors representing diverse perspectives. These individuals either head the respective national reference laboratory or lead an expert lab for the selected pathogens at BfR. Seven experts contributed to the model, including microbiologists, veterinarians, medical doctors, epidemiologists, biologists, agronomists and public health officers. They all have a proven scientific expertise in food safety, monitoring of epizootics and zoonoses, risk assessment and risk communication. We separately interviewed the experts to minimize individual biases in expert judgment, such as anchoring and availability [30], and adopted rigorous elicitation protocols for preference elicitation to reduce biases in valuation tasks [36].

### 3.2 Identification of foodborne zoonotic threats

The European Food Safety Authority (EFSA) selection of the currently known and most relevant foodborne risks [37] was the basis for our choice of threats. Seven significant threats to food supply chains in Germany were identified: *Campylobacter*, *Salmonella* (non-typhoidal), *Listeria monocytogenes*, pathogenic *Escherichia coli*, *Yersinia*, *Toxoplasma gondii*, and hepatitis E virus.

### 3.3 Definition of impact attributes

We developed four impact attributes [28] that covered the remit of the BfR for this prioritization. Each attribute was decomposed into two constructed dimensions to capture the major aspects relevant to assessing foodborne risks in Germany (modified from Del Rio Vilas et al. [14]):

1. *Public Health Impact:* Clinical outcome (severity of disease) versus the proportion of the population potentially affected (extent of damage).
2. *Economic Impact:* Branch of industry affected (severity of economic loss) versus revenue of the food sector typically affected (extent of economic damage).
3. *Social Impact:* Human perspective (consumer concerns) versus financial security (workers' interests).
4. *Consumer Perception and acceptance of foodborne diseases:* Capacity for action (potential measures) versus threat potential (awareness of the risk by consumer).

For each attribute, the expert group identified: (i) three levels of impact indicating the range of possible consequences on the first dimension; (ii) three levels of impact indicating the range of possible consequences on the second dimension; (iii) the combinations of these dimension levels generated the levels for the attribute (with the inclusion of a no impact level) as illustrated in Table 1 for the Public Health Impact attribute.

**Table 1:** Ranking and dis-value scoring (median, mean ± standard deviation) of the *Public Health Impact* of a foodborne zoonotic threat.

Clinical outcome (severity of disease)		Ranking and scoring		
	**Lethal**	**Level 5**	**Level 8**	**Level 9**
		50 (53 ± 22)	90 (88 ± 6)	100
*Hospitalization/long-term sequelae*	**Serious**	**Level 3**	**Level 6**	**Level 7**
		32 (37 ± 20)	80 (73 ± 15)	90 (86 ± 9)
*No visit to the doctor needed*	**Mild**	**Level 1**	**Level 2**	**Level 4**
		10 (10 ± 7)	20 (24 ± 13)	40 (42 ± 19)
	**Level 0** (no impact)	**Minor outbreak**	**Localized outbreak**	**Extended outbreak**
		*Local, usually a point source, like a restaurant; limited in time*	*Countrywide, several federal states are temporarily affected*	*Supraregional and/or protracted, cases are notified nationally and internationally and/or over a long period of time*
		**Proportion of the population potentially affected (extent of damage)**		

We ensured that the five desirable properties for attributes were met: unambiguous, comprehensive, direct, operational, and understandable [28]. In selecting attributes to prioritize foodborne zoonotic threats, we deliberately focused on operational and easily understandable attributes, at the expense of more detailed data acquisition that would have been required to construct fully direct and comprehensive attributes. These design trade-offs were necessary to keep the assessment rapid and readily recursive. However, we clearly specified the upper and lower limits of all attributes, which is a key requirement for its comprehensiveness, so that value trade-offs were not distorted [38]. The other three attributes were defined similarly (see Supplementary Tables A1-A4).

### 3.4 Valuation of impact levels

The valuation of each level for each impact attribute (as illustrated in Table 1 for the Public Health Impact) was conducted individually with each expert, allowing the diversity of opinions and perspectives to be preserved [36]. Initially, the experts ranked the levels within each impact attribute from worst (Level 9) to best (Level 0).

After this ordinal ranking, the experts were asked to make pairwise comparisons and to score the difference of values between each pair of levels using the direct rating method (see Montibeller [17]). The highest-ranked level (Level 9) was assigned a *dis-value* score of 100 points [18]; while the lowest-ranked level, representing no impact (Level 0), received a score of 0 points, as illustrated for the public health attribute in Table 1. The impacts for each of the three remaining attributes were valued using the same approach (see Supplementary Figs. A1-A4).

### 3.5 Elicitation of attribute weights

We elicited the prioritization of the four impact attributes from experts using the swing weighting elicitation protocol [12], adapted to the minimization of detrimental impacts [17]. Once again, we kept the definition of trade-offs at an individual level to maximize the diversity of opinions [36].

Specifically, each expert had to consider the trade-offs among the four impact attributes by considering the range of each attribute, from its maximum to its minimum level (Supplementary Fig. A5). The most relevant attribute range was assigned a score of 100 (swing weight), with the remaining attribute ranges valued proportionally relative to this reference. This protocol minimizes the range-insensitivity bias [30] by encouraging experts to consider both attribute ranges and the relative importance of each impact.

After calculating the median (as it is less sensitive to extreme valuations than the mean) from the individual swing weights provided by each expert (Supplementary Figs. A5 and A6), we normalized the swing weights by dividing each median swing weight by the sum of median swing weights. The normalized attribute weights of the Public Health, Social, Economic and Consumer Perception Impact attributes were *ω*_PH_ = 41.7%, *ω*_EC_ = 25%, *ω*_SO_ = 20.8%, and *ω*_CP_ = 12.5%, respectively. The high weight assigned to the public health attribute reflects both its importance relative to the other impact dimensions and its wide range (from *no impact* to an *extended outbreak of a lethal disease*).

## 4 Results: overall impact and expected overall impact

The results from the framework application at the BfR are presented alongside its next steps, namely the calculation of the overall impact for each threat and the subsequent assessment of its expected overall impact.

### 4.1 Calculation of the overall impact of foodborne zoonotic threats

The *deterministic* overall impact dis-value V^−^(T) for each T-th foodborne zoonotic threat is a weighted sum, with the partial dis-values v^−^_k_ of each threat on every k-th impact attribute (Public Health, Economy, Society, Consumer Perception) multiplied by the respective normalized attribute weight *ω*_*k*_ of the impact:

$$V^{-}\left(T\right)=\omega_{\mathrm{PH}}\;{v^{-}}_{\mathrm{PH}}\left(T\right)+\omega_{\mathrm{EC}}\;{v^{-}}_{\mathrm{EC}}\left(T\right)+\omega_{\mathrm{SO}}\;{v^{-}}_{\mathrm{SO}}\;\left(T\right)+\omega_{\mathrm{CP}}\;{v^{-}}_{\mathrm{CP}}\;\left(T\right)$$

**Fig. 2:**
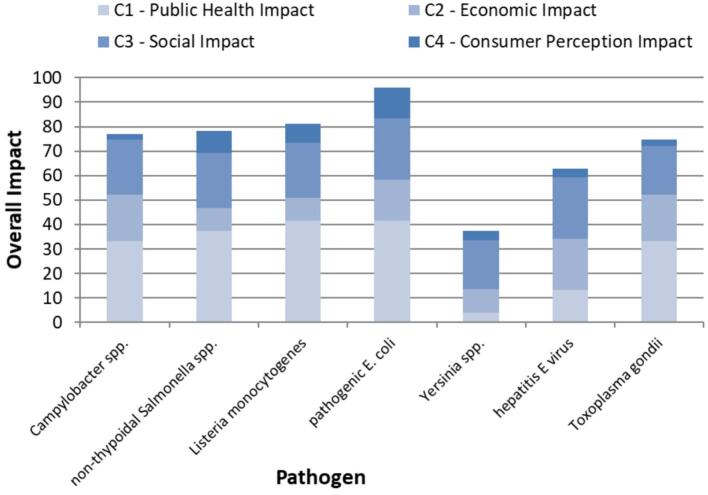
Deterministic overall impact value of the seven most relevant foodborne zoonotic threats in Germany and proportional distribution of the four impact attributes.

We calculated the *deterministic* overall impact dis-values, V^−^(T), to rank the foodborne zoonotic threats (Fig. 2). The valuation of impacts for each threat T was based on the dis-value scores for each attribute (e.g., the Public Health Impact, v^−^_PH_(T), as shown in Table 1). The higher the overall dis-value score, the higher the ranking on the priority list.

### 4.2 Prioritization of pathogen-food supply chain combinations

We estimated the probability that the pathogen is present in the food component (p_FC_) by assessing the occurrence of zoonotic threats in different food vehicles using monitoring and laboratory data. The number of pathogens isolated from food vehicles and submitted to the national reference laboratories was used as a proxy for the distribution of zoonotic pathogens across different food items (food-matrix subcategories). The proxy estimate based on laboratory data is limited to viable pathogens and is subject to selection bias, as the submitted pathogens were not necessarily representative of all contaminations across the food supply chain. Therefore, we combined the comprehensive national monitoring data and the in-house laboratory data.

We assumed that the prevalence of zoonotic pathogens in non-processed major food matrices directly correlates with their prevalence in the animal population [34]. The occurrence of the pathogen in livestock is a prerequisite for its isolation from processed animal food products (food-matrix subcategories), which in turn serves as a proxy for estimating the probability that the pathogen is present in the supply chain (p_SC_). For non-animal food products, we followed the same logic, although the occurrence of pathogens is derived from cross-contamination during food processing.

In 2019, neither the *hepatitis E virus* nor *Toxoplasma gondii* was included in the national monitoring plan for zoonoses in food. Furthermore, our institutional expert labs at BfR did not routinely collect viral or protozoan pathogens. Therefore, the prevalence rate in livestock and the detection rate in food subcategories were extracted from the scientific literature for the most relevant transmission routes identified by the experts [39], [40].

The number of food samples tested in the national monitoring program for specific foodborne pathogens varied, with *Salmonella* spp. and *Listeria monocytogenes* clearly predominating (Table 2).

**Table 2:** Number of food samples tested (positive) for zoonotic bacterial pathogens within the national monitoring of zoonoses, 2019, and number of bacterial isolates sent to reference laboratories at the German Federal Institute for Risk Assessment, 2017–2019.

Pathogen	Number of food samples tested, 2019	Number of positive test results	Percentage (%) of positive samples	Number of isolates at BfR, 2017–2019
*Campylobacter* spp.	3158	557	17.6	1634
Non-typhoidal *Salmonella* spp.	33,611	216	0.6	1352
*Listeria monocytogenes*	17,838	846	4.7	2517
Pathogenic *Escherichia coli*	3151	126	4.0	1167
*Yersinia* spp.	965	81	8.4	228

For each pathogen, we used national monitoring data on zoonotic pathogens in food (2019) and in-house laboratory information and management system (LIMS) data (2017–2019) to calculate the expected impact of each pathogen-food supply chain combination. This is exemplified in Table 3 for *Campylobacter* spp.

**Table 3:** Prevalence of major foodborne zoonotic pathogen (*Campylobacter* spp.) in food matrix categories and subcategories (retrieved from national monitoring and surveillance data on zoonoses in food collected by the food control authorities of the German federal states) and bacterial isolates extracted from food products and sent to the national reference laboratories (corresponding metadata on food matrix subcategories were retrieved from the in-house laboratory information and management system, LIMS). The publicly available food matrix catalogue published by the Federal Office of Consumer Protection and Food Safety (Bundesamt für Verbraucherschutz und Lebensmittelsicherheit, BVL) (Catalogue No. 003, Version 135.00: Matrix codes, 2018) was used to define the food matrix categories.

Pathogen	National monitoring data of zoonotic pathogens in food, 2019				In-house laboratory information and management system (LIMS), 2017‐–2019		Expected overall impact
	Affected food matrix category	Prevalence rate of a pathogen in a food matrix (%)	Main affected food matrix subcategory	Number of samples that tested positive from a specific food matrix subcategory/total number of samples that tested positive for the respective pathogen	Main affected food matrix subcategory	Number of isolates sampled from a food matrix subcategory/total number of isolates of the respective pathogen	
*Campylobacter* spp.	Meat from warm blooded animals	36.04	chicken cuts	137/557 = 0.246	chicken cuts	500/1634 = 0.306	8.50
	Meat products from warm blooded animals except for sausages	18.17	raw chicken cuts, ready to cook	58/557 = 0.104	chicken breast	270/1634 = 0.165	4.59
	Milk	1.44	stewing hen	50/557 = 0.090	turkey cuts	193/1634 = 0.118	3.28
	Eggs and egg products	1.08	chicken leg	40/557 = 0.072	chicken	103/1634 = 0.063	1.75
			chicken breast	35/557 = 0.063	chicken leg	78/1634 = 0.048	1.33

Overall impact values of the different foodborne zoonotic pathogens were multiplied by the probability of occurrence of the respective threats in various food supply chains to provide expected impacts in a pathogen-supply chain combination:

$${\mathrm{EV}}^{-}\left(T\right)=p_{\mathrm{FC}}\;p_{\mathrm{SC}}\;V^{-}\left(T\right)$$

**Fig. 3:**
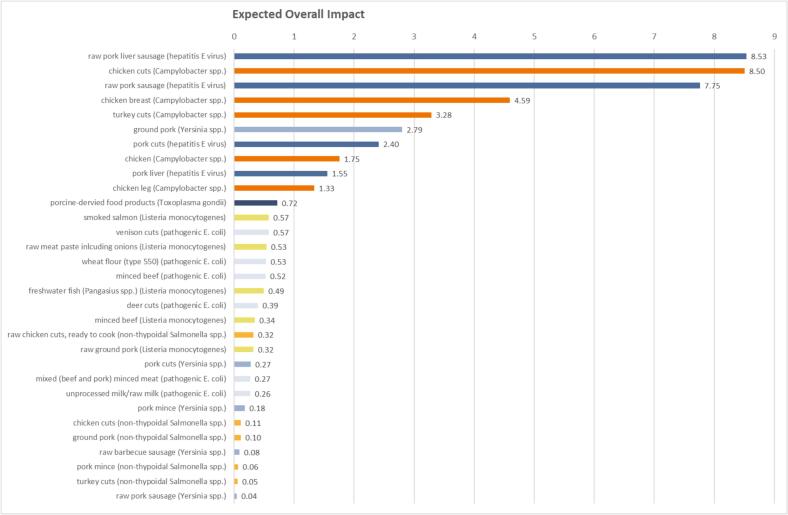
Ranking of the expected overall threat impact of the seven most relevant foodborne zoonotic pathogens in Germany in specific food products (colors indicate different pathogens).

The expected overall impact values EV^−^(T) were used to rank food-pathogen combinations, as detailed in Fig. 3 (hence chicken cuts infected by *Campylobacter* spp. are ranked 2nd with an expected overall impact of 8.5 dis-value points as detailed in Table 3).

## 5 Discussion and policy implications

In this section, we reflect on the application of our framework to prioritize foodborne zoonotic threats along food supply chains in Germany. We highlight some limitations of the evaluation and suggest ways to further improve it when applying the framework to other countries and to national and global food supply chains.

The prioritization model was able to structure a considerable amount of complex information and enabled systematic risk ranking. The experts could incorporate information from various sources, which was especially helpful for the ranking of emerging threats, where there is limited hard evidence [1]. The top-ranked threats can help identify priority actions such as stricter process controls and enhanced monitoring for relevant pathogens, as well as focused risk communication for vulnerable consumer groups, to reduce their impacts and/or the likelihood of their occurrence.

We conducted the expert elicitation individually to allow reliable judgments, independent of mainstream opinions and the institutional positions [41]. Despite the very different qualifications of BfR risk assessors, we received homogeneous judgments on the impacts of the various attribute levels.

The interviewed experts rated the value of impact reduction on the four types of impacts (Public Health, Social, Economic, and Consumer Perception) with decreasing swing weights. In contrast, policymakers may weigh impacts equally because it might be politically difficult to justify different weights. This equalizing bias [30] reduces the quality of prioritizations and should be avoided by using a rigorous elicitation protocol. Bias may also occur by neglecting relevant impacts in a specific decision context [17], which we tried to minimize by engaging with a group of relevant experts.

Since the results of our evaluation are specific to Germany, we will not discuss the priority list (Fig. 3) in detail. We note that the threats under study here were already found on a priority list of the Robert Koch Institute (RKI, the German National Public Health Institute) for Germany [13]. This study also recognized changes in priorities over time, when they compared their results with another RKI study in 2004 [16]. These previous findings are aligned with our risk-ranking results.

Other food safety or public health organizations could easily implement the evaluation model. The assessment can be applied to any other country and national or international food supply chains, if necessary, by modifying either the list of threats or the impact attributes of the evaluation model.

Although the entire food chain *from stable to table* must be assessed and controlled to eradicate zoonotic diseases in animal reservoirs and prevent human infections (following the *One Health* concept), in the present study, we focused on foodstuffs as sources of pathogens because they directly lead to exposure through consumption. The probability of a zoonotic pathogen occurring in specific food matrices substantially altered our risk-ranking list. For example, while the overall impact of hepatitis E virus itself was quite low (ranked sixth out of seven threats; Fig. 2), the expected overall impact of hepatitis E virus in raw pork liver sausages was the highest risk in our study (Fig. 3). However, no food samples were tested for hepatitis E virus up to the time of our study, questioning the efficiency of the current monitoring and surveillance plan in Germany.

Risk-ranking exercises should be repeated periodically to consider changes in disease drivers and to refine the knowledge base for each decision in the context of the current situation [15]. Interim assessments may also be necessary in the event of (re-)emerging threats. Recurrent iterations of the decision modeling process with the same group of experts will sharpen results for future applications and strengthen strategies to mitigate zoonotic threats in food supply chains.

In multi-stakeholder situations, often present in food safety decisions, this evaluation framework may facilitate the coordination of different interests and the exchange of ideas and knowledge between scientists and policymakers, which is essential for risk-based and cost-effective surveillance of foodborne threats [42]. This effective management of food supply chain risks can help improve national and global food safety. The benefit of reducing the impacts or likelihood of outbreaks for each threat can be quantified and compared, thereby identifying the optimal portfolio of risk-reduction actions that maximizes the value for money of the investment [17].

Another advantage is that our model can be easily adapted to other infectious diseases (through de novo assessment of impacts by appropriate experts) and other countries (by ranking and scoring pathogens relevant in the geographic region under study). The threat ranking assessment can be easily or automatically updated when new data are available, making this application potentially helpful in analyzing crisis scenarios and for recursive prioritizations [2], [17].

## CRediT authorship contribution statement

**Sascha Al Dahouk:** Writing – review & editing, Writing – original draft, Visualization, Validation, Project administration, Methodology, Investigation, Formal analysis, Data curation, Conceptualization. **Gilberto Montibeller:** Writing – review & editing, Writing – original draft, Validation, Supervision, Project administration, Methodology, Data curation, Conceptualization.

## Declaration of competing interest

The authors declare that they have no known competing financial interests or personal relationships that could have appeared to influence the work reported in this paper.

## Data Availability

Data will be made available on request.

## Appendix A Supplementary data

Supplementary material Image 1
