# Supplementary material for: A framework for the recursive risk-ranking of foodborne zoonotic threats along food supply chains
Source: One Health. 2026 Apr 15;22:101415. doi: 10.1016/j.onehlt.2026.101415 (PMC13103578; doi:10.1016/j.onehlt.2026.101415)
Supplement: Supplementary file 1 — Supplementary material [file mmc1.pdf]

S. Al Dahouk and G. Montibeller, A framework for the recursive risk-ranking of foodborne zoonotic threats along food supply chains, *One Health* (2026), https://doi.org/10.1016/j.onehlt.2026.101415

Supplementary Material Online: A Framework for the Recursive Risk-Ranking of Foodborne Zoonotic Threats along Food Supply Chains – Evaluation Model

Table A1. Ranking and dis-value scoring (median, mean ± standard deviation) of the *public health impact* of a foodborne zoonotic hazard.

**Clinical outcome (severity of disease)**		**Ranking and scoring**		
	**lethal**	**Level 5**<br>50 (53 ± 22)	**Level 8**<br>90 (88 ± 6)	**Level 9**<br>100
hospitalization/long-term sequelae	**serious**	**Level 3**<br>32 (37 ± 20)	**Level 6**<br>80 (73 ± 15)	**Level 7**<br>90 (86 ± 9)
no visit to the doctor needed	**mild**	**Level 1**<br>10 (10 ± 7)	**Level 2**<br>20 (24 ± 13)	**Level 4**<br>40 (42 ± 19)
	**Level 0**<br>(no impact)	**minor outbreak**	**localized outbreak**	**extended outbreak**
		local, usually a point source, like a restaurant; limited in time	*countrywide, several federal states are temporarily affected*	*supraregional and/or protracted, cases are notified nationally and internationally and/or over a long period of time*
		**Proportion of the population potentially affected (extent of damage)**		

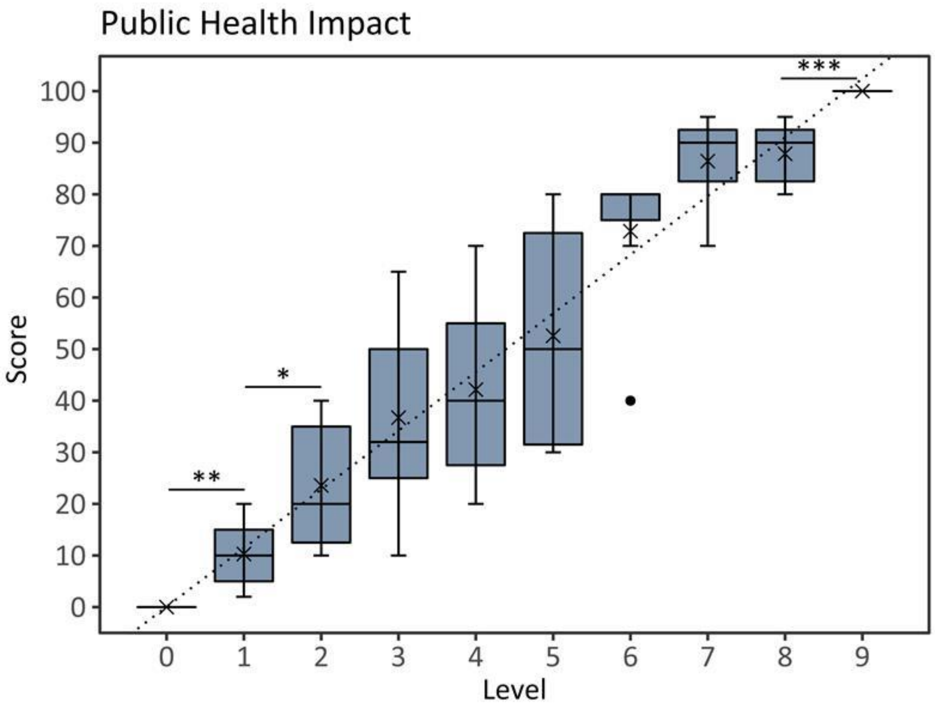

Figure A1. Boxplot graph of the dis-value scores at different *public health* impact levels (mean, median, upper and lower quartile, minimum and maximum values as well as outliers are presented). The dotted line shows the linear regression. Asterisks illustrate the two-sided significance level resulting from the t-test, with *p ≤ 0.05, **p ≤ 0.01, and ***p ≤ 0.001.

Table A2. Ranking and dis-value scoring (median, mean ± standard deviation) of the *economic impact* of a foodborne zoonotic hazard (economic loss).

**Branch of industry affected (severity of economic loss)**		**Ranking and scoring**		
from stable to table	**total value chain of a food sector**	**Level 4**<br>40 (47 ± 11)	**Level 7**<br>80 (77 ± 8)	**Level 9**<br>100
animal health, ban on trade, culling, rural business	**livestock/agricultural industry**	**Level 3**<br>30 (34 ± 10)	**Level 6**<br>60 (54 ± 14)	**Level 8**<br>90 (77 ± 17)
food production plants, primary producers, retailers	**small and medium-sized enterprises**	**Level 1**<br>10 (20 ± 15)	**Level 2**<br>25 (34 ± 19)	**Level 5**<br>45 (50 ± 22)
	**Level 0** (no impact)	**low**	**medium**	**large**
		others, such as fish and eggs	*fruits and vegetables*	*meat, dairy and pasta products*
		**Revenue of the food sector typically affected (extent of economic damage)**		

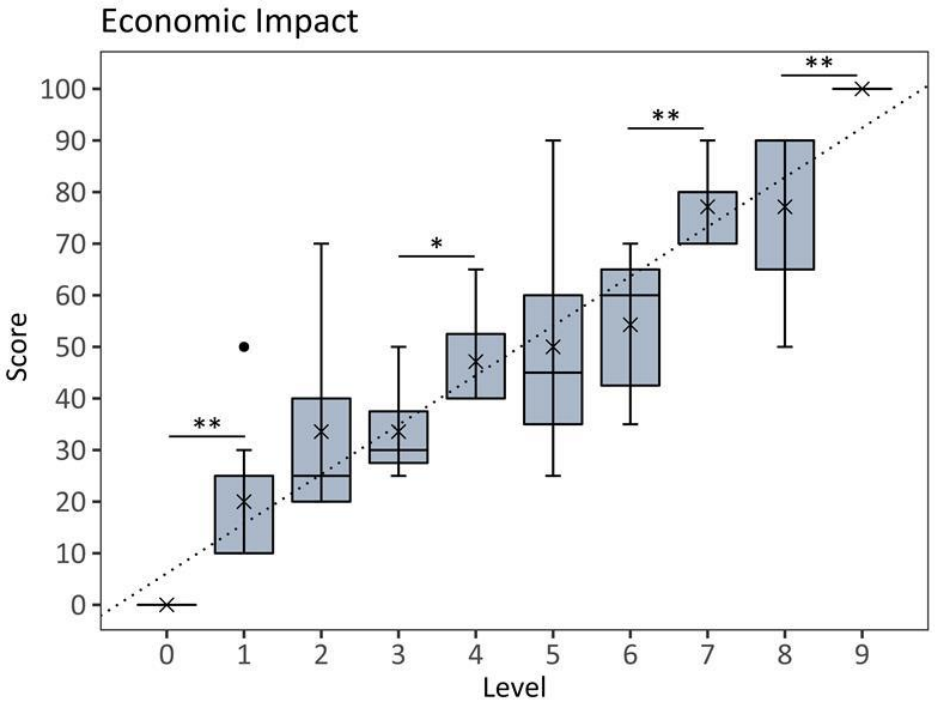

Figure A2. Boxplot graph of the dis-value scores at different *economic* impact levels (mean, median, upper and lower quartile, minimum and maximum values as well as outliers are presented). The dotted line shows the linear regression. Asterisks illustrate the two-sided significance level resulting from the t-test, with $*p \leq 0.05$ and $**p \leq 0.01$.

Table A3. Ranking and dis-value scoring (median, mean ± standard deviation) of the *social impact* of a foodborne zoonotic hazard (political concerns).

**Human perspective (consumer concerns)**		**Ranking and scoring**		
elderly, infants and young children, pregnant women, immunocompromised are mainly affected	**high**	**Level 6**<br>80 (75 ± 13)	**Level 8**<br>90 (89 ± 5)	**Level 9**<br>100
low-income consumers, single mothers, pensioners are mainly affected	**medium**	**Level 3**<br>60 (56 ± 9)	**Level 5**<br>70 (72 ± 7)	**Level 7**<br>80 (83 ± 8)
marginalized subpopulations, experiencing discrimination and exclusion are mainly affected	**low**	**Level 1**<br>40 (36 ± 16)	**Level 2**<br>50 (49 ± 20)	**Level 4**<br>60 (62 ± 18)
	**Level 0**<br>(no concern)	**minor source of employment**	**medium source of employment**	**large source of employment**
		small enterprises, <50 employees, annual revenue <10 Mill Euros	*medium-sized enterprises, 50-250 employees, annual revenue 10-50 Mill Euros*	*large enterprises, >250 employees, annual revenue >50 Mill Euros*
		**Financial security (workers' interests)**		

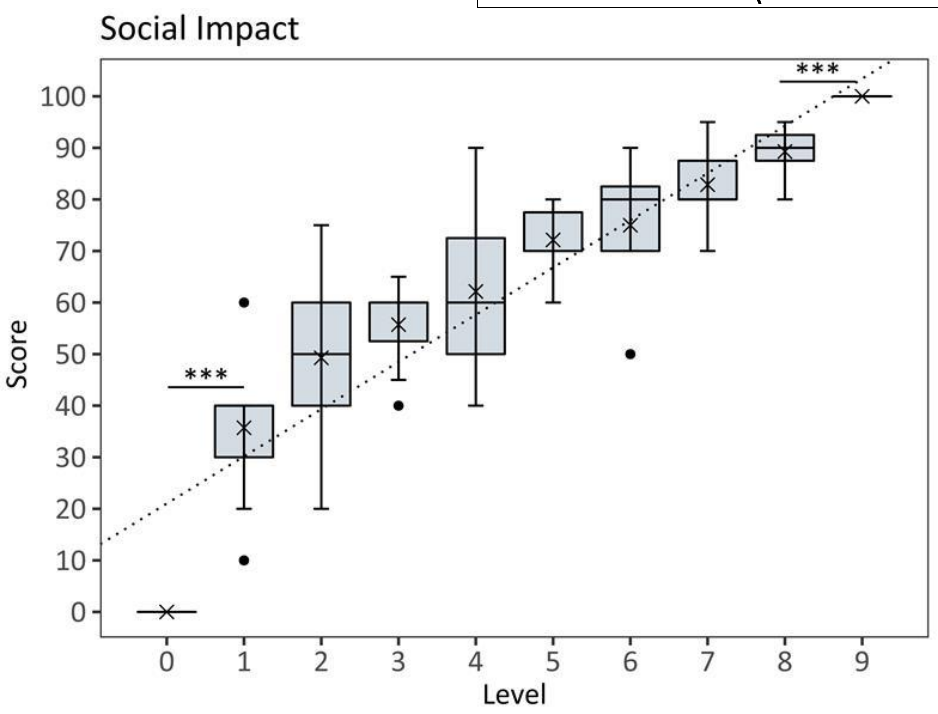

Figure A3. Boxplot graph of the dis-value scores at different *social* impact levels (mean, median, upper and lower quartile, minimum and maximum values as well as outliers are presented). The dotted line shows the linear regression. Asterisks illustrate the two-sided significance level resulting from the t-test, with ***p ≤ 0.001.

Table A4. Ranking and dis-value scoring (median, mean ± standard deviation) of the *consumer perception impact* of a foodborne zoonotic hazard.

**Capacity for action (potential measures)**		**Ranking and scoring**		
	**risk is uncontrollable**	**Level 5**<br>50 (39 ± 21)	**Level 8**<br>90 (87 ± 9)	**Level 9**<br>100
e.g. HACCP concepts are in place, surveillance and control measures	**risk is managed by food producer or can be controlled by public authorities**	**Level 3**<br>30 (26 ± 15)	**Level 6**<br>60 (60 ± 14)	**Level 7**<br>70 (71 ± 11)
e.g. by proper food processing and kitchen hygiene measures	**risk is controllable by consumer**	**Level 1**<br>10 (8 ± 7)	**Level 2**<br>20 (23 ± 10)	**Level 4**<br>30 (32 ± 13)
	**Level 0**<br>(no exposure/no risk)	**exposure to risk is known and accepted**	**exposure to risk is known, but involuntary**	**potential exposure is unknown**
		**Hazard potential (awareness of the risk by consumer)**		

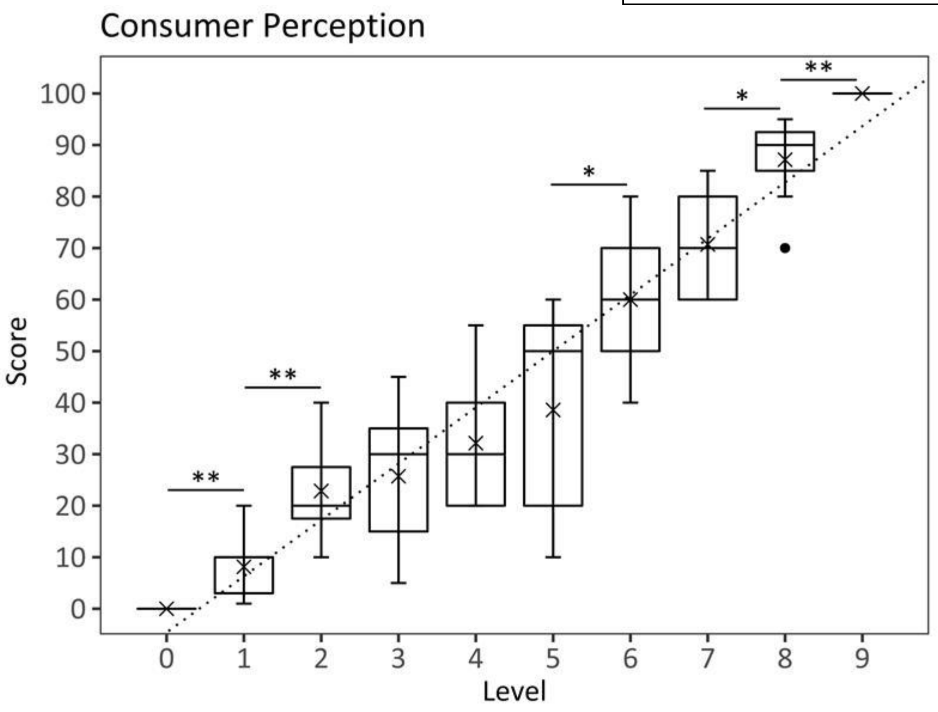

Figure A4. Boxplot graph of the dis-value scores at different *consumer perception* impact levels (mean, median, upper and lower quartile, minimum and maximum values as well as outliers are presented). The dotted line shows the linear regression. Asterisks illustrate the two-sided significance level resulting from the t-test, with *p ≤ 0.05 and **p ≤ 0.01.

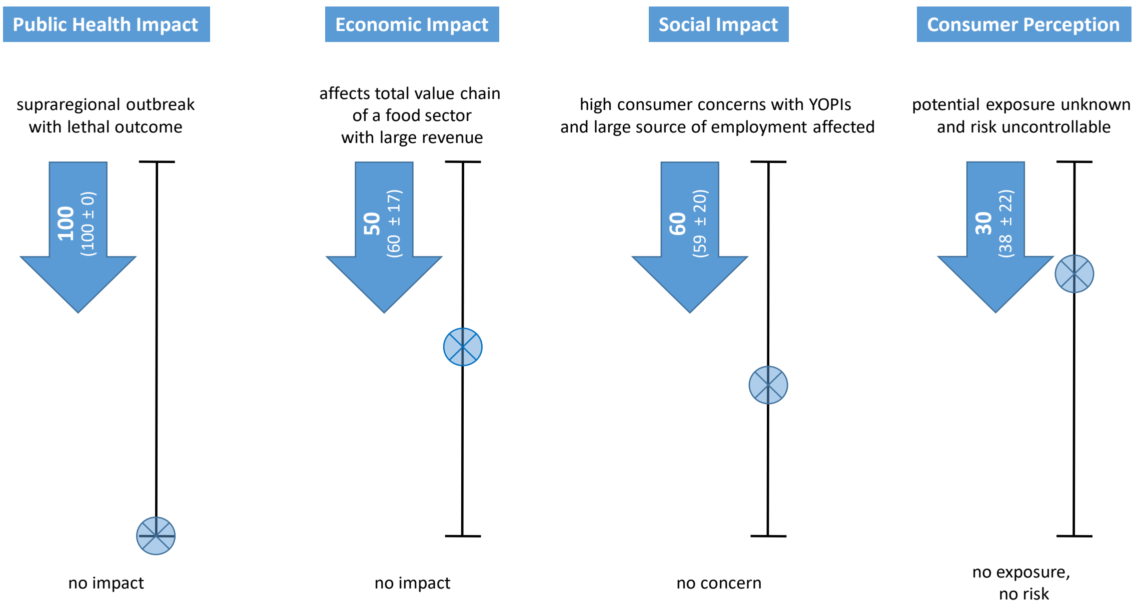

Figure A5. Elicitation of swing weights by considering the range of each attribute, from its maximum (Level 9) to its minimum level (Level 0) (median swing weight (mean ± standard deviation)).

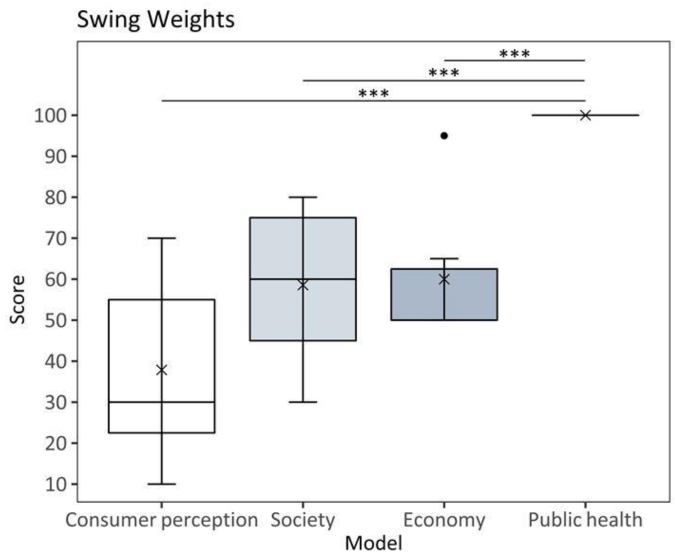

Figure A6. Boxplot graph of the swing weights elicited from food safety experts for the four impacts (mean, median, upper and lower quartile, minimum and maximum values as well as outliers are presented). Asterisks illustrate the two-sided significance level resulting from the t-test, with ***p ≤ 0.001.
